# Perceived frailty and measured frailty among adults undergoing hemodialysis: a cross-sectional analysis

**DOI:** 10.1186/s12877-015-0051-y

**Published:** 2015-04-24

**Authors:** Megan L Salter, Natasha Gupta, Allan B Massie, Mara A McAdams-DeMarco, Andrew H Law, Reside Lorie Jacob, Luis F Gimenez, Bernard G Jaar, Jeremy D Walston, Dorry L Segev

**Affiliations:** Department of Epidemiology, Johns Hopkins University School of Public Health, Baltimore, MD USA; Johns Hopkins University Center on Aging and Health, Baltimore, MD USA; Department of Surgery, Johns Hopkins University School of Medicine, 720 Rutland Ave, Ross 36, Baltimore, MD 21205 USA; Welch Center for Prevention, Epidemiology and Clinical Research, Baltimore, MD USA; Center for Health Systems Effectiveness, Oregon Health and Science University, Portland, OR USA; Department of Medicine, Johns Hopkins University School of Medicine, 1830 Building Room 416 Nephrology, 600 North Wolfe Street, Baltimore, MD 21287 USA; Nephrology Center of Maryland, 5601 Loch Raven Boulevard, Suite 3 North, Baltimore, MD 21239 USA; Department of Geriatric Medicine and Gerontology, Johns Hopkins University School of Medicine, 5501 Hopkins Bayview Circle, Room 1A.62, Baltimore, MD 21224 USA; Johns Hopkins Asthma and Allergy Center, 5501 Hopkins Bayview Cir, Baltimore, MD 21224 US

**Keywords:** Frailty, Dialysis, End-stage renal disease

## Abstract

**Background:**

Frailty, a validated measure of physiologic reserve, predicts adverse health outcomes among adults with end-stage renal disease. Frailty typically is not measured clinically; instead, a surrogate—perceived frailty—is used to inform clinical decision-making. Because correlations between perceived and measured frailty remain unknown, the aim of this study was to assess their relationship.

**Methods:**

146 adults undergoing hemodialysis were recruited from a single dialysis center in Baltimore, Maryland. Patient characteristics associated with perceived (reported by nephrologists, nurse practitioners (NPs), or patients) or measured frailty (using the Fried criteria) were identified using ordered logistic regression. The relationship between perceived and measured frailty was assessed using percent agreement, kappa statistic, Pearson’s correlation coefficient, and prevalence of misclassification of frailty. Patient characteristics associated with misclassification were determined using Fisher’s exact tests, t-tests, or median tests.

**Results:**

Older age (adjusted OR [aOR] = 1.36, 95%CI:1.11-1.68, P = 0.003 per 5-years older) and comorbidity (aOR = 1.49, 95%CI:1.27-1.75, P < 0.001 per additional comorbidity) were associated with greater likelihood of nephrologist-perceived frailty. Being non-African American was associated with greater likelihood of NP- (aOR = 5.51, 95%CI:3.21-9.48, P = 0.003) and patient- (aOR = 4.20, 95%CI:1.61-10.9, P = 0.003) perceived frailty. Percent agreement between perceived and measured frailty was poor (nephrologist, NP, and patient: 64.1%, 67.0%, and 55.5%). Among non-frail participants, 34.4%, 30.0%, and 31.6% were perceived as frail by a nephrologist, NP, or themselves. Older adults (P < 0.001) were more likely to be misclassified as frail by a nephrologist; women (P = 0.04) and non-African Americans (P = 0.02) were more likely to be misclassified by an NP. Neither age, sex, nor race was associated with patient misclassification.

**Conclusions:**

Perceived frailty is an inadequate proxy for measured frailty among patients undergoing hemodialysis.

## Background

Frailty is a phenotype of poor physiologic reserve, multisystem dysregulation, and increased vulnerability to stressors [1]. While much of the early research on frailty occurred in populations of older adults [1-4], this validated measure is gaining importance among those with chronic conditions, such as end-stage renal disease (ESRD) [5]. As in older adult populations [1,4], being frail is predictive of falls [6], hospitalization and mortality [5], among patients with ESRD, irrespective of age. Furthermore, frailty is predictive of delayed graft function [7], early hospital readmission [8], and mortality [9] after kidney transplantation.

Despite strong associations with poor outcomes for patients with ESRD, frailty is not routinely assessed clinically [10]; as such, clinical assessments of decreased physiologic reserve and vulnerability to stressors tend to be based on a combination of a provider’s clinical experience, provider perceptions of patient frailty, and patient perception of their own frailty, rather than a validated measure [2]. Moreover, the common perception that older adults and women generally tend to be more frail [11] may impact clinical decision-making such as choices about renal replacement therapies. Indeed, older adults and women have lower access to transplantation [12-17] even among appropriate transplant candidates [18].

Whether perceptions about frailty accurately reflect measured frailty remains unknown. To better understand the relationship between perceived and measured frailty, we sought 1) to assess and compare patient characteristics associated with measured, provider-perceived, and patient-perceived frailty, 2) to compare provider- and patient-perceived frailty with measured frailty, and 3) to identify patient characteristics associated with misclassification of frailty status.

## Methods

### Study population

In this cross-sectional study, 146 adults undergoing hemodialysis were recruited between January 2009 and March 2010 from a single dialysis center in Baltimore, Maryland. Inclusion criteria included: age ≥18 years and English-speaking. Because of the high prevalence of frailty among younger and older patients with ESRD, and because frailty is predictive of poor health outcomes in patients of all ages with renal disease [5,8], we included adults of all ages. All participants provided written informed consent, and all study procedures were approved by the Johns Hopkins Medical Institutions Institutional Review Board.

### Participant characteristics

Demographics (age, sex, race, education, employment, and marital status), household size, smoking history, and time on dialysis were obtained through participant self-report. Body mass index (BMI) was calculated using measured height and dry weight with obesity defined as a BMI ≥ 30. Comorbidities were abstracted from medical records and included hypertension, diabetes, peripheral vascular disease, congestive heart failure, myocardial infarction, angina pectoris, chronic obstructive pulmonary disease, history of cancer, and rheumatoid arthritis. Disability was determined using participant-reported difficulties with six activities of daily living (ADLs) including: bathing, toileting, dressing, grooming, eating, and ambulation [19].

### Measured frailty

The five components of frailty as defined by Fried *et al.* were measured: 1) shrinking (self-report of unintentional weight loss of more than 10 pounds in the past year based on dry weight, i.e. the weight of an individual undergoing hemodialysis without the excess fluid that accrues between dialysis treatments); 2) weakness (grip-strength below an established cut-off based on sex and BMI); 3) exhaustion (self-report); 4) low activity (kcal/week below an established cut-off); and 5) slow walking speed (time needed to walk 15 feet below an established cutoff based on sex and height) [1,3]. A frailty score was calculated as the number of frailty components reported for an individual (range 0–5) and categorized as non-frail (0–1 components), intermediately frail (2 components), and frail (3–5 components). As previously described [5], this categorization maintained Fried’s definition of frailty, but expanded non-frail to include a score of 1 to account for the small number (7%) of participants with a frailty score of 0.

### Perceived frailty

Perceived frailty for each participant was assessed in three ways: 1) nephrologist-perceived frailty, 2) nurse practitioner (NP)-perceived-frailty, and 3) patient-perceived frailty. Nephrologists (N = 9) and NPs (N = 4) were informed that frailty is a syndrome characterized by a loss of physiologic reserve and assessed using the five components described above and then were asked to categorize their patients as non-frail, intermediately frail, or frail. Participants were asked, “how frail do you think you are?” and were asked to categorize themselves in the same manner.

### Participant characteristics and frailty

Univariate and multivariable ordinal logistic regression models were used to estimate the log odds of measured or perceived frailty associated with various characteristics. The functional form of age was determined empirically to be continuous, as was the functional form of number of comorbidities and disability based on number of ADLs. Multivariable models included participant characteristics that were selected based on statistical significance or *a priori* rationale. Each participant was rated by one nephrologist and one NP. However, because each nephrologist and NP rated more than one participant, standard errors were estimated allowing for intragroup correlation.

### Relationship between measured and perceived frailty

The relationship between measured and perceived frailty was assessed using percent agreement, and a weighted kappa statistic was calculated. Additionally, the correlation between measured and perceived frailty was determined using Pearson’s correlation coefficient.

### Participant characteristics and misclassification

Prevalences of misclassification of frailty status by nephrologists, NPs, and patients were determined. Participant characteristics among those misclassified as intermediately frail or frail were compared to those correctly classified as non-frail using Fisher’s exact test for categorical variables, t-tests for pseudonormally distributed continuous variables, or Hodges-Lehmann’s test for equal medians for non-normally distributed continuous variables.

### Statistical analysis

All analyses were performed using STATA 12.1/SE (College Station, Texas).

## Results

### Study population

Of 146 participants, the median age was 61 years (IQR: 53, 70), 46.6% were women, 84.3% were African American, the median number of comorbidities was 3 (IQR: 2, 4), and the median number of ADLs for which participants reported difficulty was 0 (IQR: 0, 1). The median time on dialysis was 3.6 years (IQR: 1.4-6.4) (Table 1).

**Table 1:** **Participant characteristics: patients undergoing hemodialysis in a single center in Baltimore (n = 146)**

	***%*** ^**a**^ ***, Total (n = 146)***
Age (years), median [IQR]	61 [53, 70]
Women	46.6
African American race	84.3
Post-secondary education	25.4
Currently employed	8.9
Marital status	
Married/cohabitating	34.7
Single	28.5
Separated/divorced	18.8
Widowed	18.1
Lives with children	30.8
History of smoking	21.2
BMI, median [IQR]	26.7 [23.0, 33.5]
No. comorbidities, median [IQR]	3 [2,4]
Hypertension	89.0
Diabetes	65.8
Peripheral vascular disease	30.1
Congestive heart failure	39.0
Myocardial infarction	16.4
Angina pectoris	4.8
COPD	19.2
History of cancer	18.5
Rheumatoid arthritis	6.9
Number of ADLs, median [IQR]	0 [0, 1]
Time on dialysis (years), median [IQR]	3.6 [1.4, 6.4]

Abbreviations: *IQR* interquartile range, *BMI* body mass index, *COPD* chronic obstructive lung disease, *ADLs* activities of daily living.

^a^All values are percentages unless otherwise indicated.

### Participant characteristics and frailty

In multivariable models, only disability was associated with measured frailty (adjusted OR [aOR] = 1.47, 95% CI: 1.04-2.08, P = 0.03 for each additional ADL difficulty). In contrast, age (aOR = 1.36, 95% CI: 1.11-1.68, P = 0.003 per 5-year increase in age), smoking (aOR = 3.05, 95% CI: 1.28-7.29, P = 0.01), obesity (aOR = 0.21, 95% CI: 0.16-0.29, P < 0.001, and comorbidity (aOR = 1.49, 95% CI: 1.27-1.75, P < 0.001 per one additional comorbidity) were associated with nephrologist-perceived frailty. Being non-African American (aOR = 5.51, 95% CI: 3.21-9.48, P = 0.003), being intermediately frail (aOR = 6.23, 95% CI: 2.35-16.5, P < 0.001), being employed currently (aOR = 0.16, 95% CI: 0.05-0.53, P = 0.003), having a post-secondary education (aOR = 0.37, 95% CI: 0.31-0.45, p < 0.001), and being obese (aOR = 0.44, 95% CI: 0.27-0.72, P = 0.001) were associated with NP-perceived frailty. Being non-African American (aOR = 4.20, 95% CI: 1.61-10.9, P = 0.003), smoking (aOR = 3.69, 95% CI: 1.54-8.81, P = 0.01), disability (aOR = 1.43, 95% CI: 1.02-1.99, P < 0.04 for each additional ADL difficulty), and being older (aOR = 0.81, 95% CI: 0.70-0.95, P < 0.001) were associated with patient-perceived frailty (Table 2).

**Table 2:** **Multivariable associations between participant characteristics and measured or perceived frailty**

	**Odds ratio (95% CI)** ^**a**^			
	***Measured frailty***	***Nephrologist-perceived frailty***	***NP-perceived frailty***	***Patient-perceived frailty***
Age (per 5-yr increase)	1.08 (0.95-1.24)	**1.36** (1.11-1.68)	1.07 (0.76-1.51)	**0.81** (0.70-0.95)
Being a woman	1.34 (0.71-2.53)	1.74 (0.91-3.31)	2.25 (0.82-6.21)	1.18 (0.57-2.42)
Non-African American race	1.72 (0.71-4.17)	1.30 (0.79-2.16)	**5.51** (3.21-9.48)	**4.20** (1.61-10.9)
Post-secondary education			**0.37** (0.31-0.45)	
Currently employed			**0.16** (0.05-0.53)	
History of smoking		**3.05** (1.28-7.29)		**3.69** (1.54-8.81)
Obese	1.27 (0.65-2.47)	**0.21** (0.16-0.29)	**0.44** (0.27-0.72)	
Comorbidities^b^	1.07 (0.83-1.38)	**1.49** (1.27-1.75)	1.23 (0.73-2.06)	1.29 (0.97-1.72)
Disability^c^	**1.47** (1.04-2.08)	1.26 (0.70-2.24)	1.97 (0.63-6.18)	**1.43** (1.02-1.99)
Frailty				
Non frail	-	ref	ref	ref
Intermediately frail	-	1.21 (0.53-2.78)	**6.23** (2.35-16.5)	2.06 (0.79-5.36)
Frail	-	2.77 (0.87-8.83)	4.19 (0.92-19.1)	1.28 (0.50-3.30)

Abbreviations: *NP* Nurse Practitioner, *CI* confidence interval.

^a^95% CIs for the for neprhologist-perceived frailty and NP-perceived frailty were estimated allowing for intragroup correlation because the same nephrologist or NP was able to rate multiple participants.

^b^per one comorbidity increase.

^c^per reported increase in difficulty with one activity of daily living (ADL).

Bold indicates significance at the P<0.05 level.

Odds ratios were estimated using ordered logistic regression with an order of non-frail, intermediately frail, and frail. Under the proportional odds assumption, the estimated odds ratio applies to either of the two odds ratios being modeled: for example, participants who reported difficulty with one ADL were 1.47-fold more likely to be intermediately frail or frail compared to non-frail and 1.47-fold more likely to be frail compared to intermediately frail or non-frail relative to participants who reported no difficulty for any ADL.

### Agreement between measured and perceived frailty

Among frail participants, only 42.0% and 39.2% were correctly perceived as frail by their nephrologist or NP, and only 4.9% perceived themselves as frail. Among non-frail participants, 34.4%, 30.0%, and 31.6% were incorrectly perceived as intermediately frail or frail by a nephrologist, NP, and themselves, respectively. The agreement between measured and perceived frailty was, at best, only slightly better than what would be expected by chance alone (nephrologists: 64.1% observed agreement vs. 52.9% expected agreement, kappa = 0.24; NPs: 67.0% observed agreement vs. 54.5% expected agreement, kappa = 0.27; patients: 55.5% observed agreement vs. 52.4% expected agreement, kappa = 0.07) (Table 3).

**Table 3:** **Relationships between measured and perceived frailty**

**Perceived frailty**	**Measured frailty**					
	**Non-frail, n (%)**	**Intermediately frail, n (%)**	**Frail, n (%)**	**Percent agreement**	**Kappa**	**Correlation**
Nephrologist^a^						
Non-frail	21 (65.6)	20 (51.3)	15 (30.0)	64.1	0.24	0.32
Intermediately frail	7 (21.9)	11 (28.1)	14 (28.0)			
Frail	4 (12.5)	8 (20.5)	21 (42.0)			
Nurse Practitioner^b^						
Non-frail	21 (70.0)	9 (26.5)	11 (21.6)	67.0	0.27	0.35
Intermediately frail	5 (16.7)	13 (38.2)	20 (39.2)			
Frail	4 (13.3)	12 (35.3)	20 (39.2)			
Patient^c^						
Non-frail	26 (68.4)	23 (48.9)	32 (52.5)	55.5	0.07	0.09
Intermediately frail	10 (26.3)	21 (44.7)	26 (42.6)			
Frail	2 (5.3)	3 (6.4)	3 (4.9)			
**Nurse practitioner** ^**b**^	**Nephrologist** ^**a**^ **perceived frailty** ^**d**^					
**Perceived frailty**	**Non-frail, n (%)**	**Intermediately frail, n (%)**	**Frail, n (%)**	**Percent agreement**	**Kappa**	**Correlation**
Non-frail	25 (51.0)	10 (32.3)	5 (16.7)	64.6	0.21	0.28
Intermediately frail	14 (28.6)	10 (32.3)	13 (43.3)			
Frail	10 (20.4)	11 (35.5)	12 (40.0)			

^a^Of the 146 participants with measured frailty, 121 were rated by a nephrologist.

^b^Of the 146 participants with measured frailty, 115 were rated by a nurse practitioner.

^c^Of the 146 participants with measured frailty, 146 provided self-rated frailty.

^d^Of the 146 participants, 110 participants were rated by both a nephrologist and a nurse practitioner.

Percents sum down columns. Thus, among participants who were non-frail based on measured frailty, 21.9% and 12.5% were misclassified as intermediately frail and frail by a nephrologist.

### Participant characteristics and misclassification

Among those who were non-frail according to measured frailty, those misclassified as frail or intermediately frail by a nephrologist did not differ by sex (P = 0.28) or race (P = 0.27), but they were statistically significantly older (mean age of those misclassified of 67.5 vs. 47.0 of those not misclassified, P < 0.001). In contrast, those misclassified by an NP were more likely to be women (66.7% of those misclassified vs. 23.8% of those not misclassified, P = 0.04) or non-African American (33.3% of those misclassified vs. 0% of those not misclassified, P = 0.02); they were clinically, but not statistically significantly, older (mean age of those misclassified of 62.8 vs. 53.0 of those not misclassified, P = 0.09). Those who misclassified themselves did not differ by sex (P = 0.73), race (P = 0.58), or age (P = 0.83). None of the other participant characteristics (education, employment, smoking history, obesity, comorbidities, or disability) were associated with being misclassified as intermediately frail or frail (Table 4).

**Table 4:** **Participant characteristics by misclassification of frailty status among non-frail participants**

	**Nephrologist-perceived frailty, n = 32**		**P**	**NP-perceived frailty, n = 30**		**P**	**Patient-perceived frailty, n = 38**		**P**
	**non-frail**	**int. frail/frail**		**non-frail**	**int. frail/frail**		**non-frail**	**int. frail/frail**	
Age (years)^a^	47.0	67.5	<0.001	53.0	62.8	0.09	54.8	55.8	0.83
Women	33.3	54.6	0.28	23.8	66.7	0.04	38.5	50.0	0.73
Non-African American race	4.8	18.2	0.27	0.0	33.3	0.02	7.7	16.7	0.58
Post-secondary education	23.8	27.3	1.00	28.6	22.2	1.00	21.7	27.3	0.76
Currently employed	19.1	18.2	1.00	23.8	0	0.29	21.7	9.1	0.31
History of smoking	19.1	9.1	0.64	19.1	11.1	1.00	13.0	22.7	0.32
Obese	33.3	27.3	1.00	38.1	22.2	0.68	30.4	27.3	1.00
Comorbidities^b^	2.3	3.2	0.07	2.4	3.0	0.27	2.5	2.7	0.56
Disability^c^	0	0	0.48	0	0	0.35	0	0	0.11

Abbreviations: *NP* nurse practitioner, *Int. frail* intermediately frail.

^a^Mean.

^b^Median number of comorbidities.

^c^Median number of activities of daily living with which participant reported difficulty.

P-values were estimated using Fisher’s exact test for categorical variables, t-tests for pseudonormally distributed continuous variables, or Hodges-Lehmann’s test for equal medians for non-normally distributed continuous variables.

## Discussion

In this cross-sectional study of adults undergoing hemodialysis, perceived frailty appeared to be an inadequate proxy for measured frailty, with fewer than half of frail patients correctly classified by themselves, their NPs, or their nephrologists. Perceived frailty according to nephrologists, NPs, and patients agreed with measured frailty only slightly better than what would be expected by chance alone. Moreover, participant characteristics associated with misclassification of frailty status as well as perceived frailty varied depending on who rated frailty status (nephrologist, NP, or patient) and differed from participant characteristics associated with measured frailty.

While other studies have explored differences between patient and provider perceptions of health status [20,21], our study compared provider and patient perceptions of frailty to a validated measure. Our novel finding that perceived and measured frailty have poor correlation provides evidence that providers and patients are inaccurate in assessing physiologic reserve and ability to respond to stressors in the same manner as a validated measure of frailty. Further, the discordance between providers’ perceptions of frailty and measured frailty may be even greater in practice because providers in this study were informed of the criteria included in measured frailty. Additionally, our findings suggest that certain groups of patients (e.g. older adults, non-African Americans, and women) are more likely to be incorrectly perceived as frail. Although others suggest a potential relationship between perceived frailty and survival [22], whether such misclassification could influence clinical decisions for treatment courses remains unclear. Furthermore, because patients of all ages were misclassified, assessing the frailty status of younger and older adults using objective criteria in the setting of chronic disease may have clinical value.

Interestingly, older participants in our study were less likely to perceive themselves as frail. Similarly, older individuals are less likely to perceive their overall health status as poor, even as their health declines [23]. One possible explanation is that older adults with ESRD compare themselves to their peers of similar age who are more likely to have other chronic health conditions. Thus, unlike younger patients with ESRD who are more likely to have healthy peers of similar age, the difference in health status between older adults with and without ESRD may not be as great, and older adults with ESRD may perceive themselves as equally healthy relative to their peers.

This study has several limitations. First, participants were drawn from a small sample of patients at a single dialysis center, limiting generalizability and statistical power to detect small effect sizes. However, with such a major discordance between measured and perceived frailty, even a much larger study is unlikely to identify agreement. Second, the sampling strategy may induce prevalent sampling bias in which our results are only generalizable to those who become prevalent dialysis patients and not to those who initiate dialysis but do not live long enough to enroll in a study. While this sampling might limit generalizability, it does not affect internal validity, as the discordance between measured and perceived frailty did not change based on time-on-dialysis. Finally, while our finding that measured and perceived frailty are poorly correlated is interesting, and while measured frailty is a validated predictor of outcomes in ESRD patients, a larger study that examines the association between perceived frailty and outcomes would better inform the clinical relevance of our findings. This study also has several strengths. The novel collection of a validated, measured frailty construct in conjunction with patient- and provider-perceived frailty allowed ascertainment of discordance between measured and perceived frailty, which to our knowledge has not been studied previously.

## Conclusions

In conclusion, provider and patient perceptions of frailty were not accurately reflective of measured frailty in a population of patients of all ages undergoing hemodialysis. Furthermore, participant characteristics associated with perceived frailty varied according to nephrologist, NP, and patients and were not consistent with participant characteristics associated with measured frailty. Notably, older adults and women were more likely to be misclassified as frail and are less likely to receive a transplant [12-18]. Thus, the impact of perceived frailty on clinical-decision making and patient outcomes warrants further investigation, as perceptions may influence patient and provider behaviors.

## Abbreviations

ADLs: Activities of daily living
aOR: Adjusted odds ratio
BMI: Body mass index
CI: Confidence interval
ESRD: End-stage renal disease
IQR: Interquartile range
NP: Nurse practioner
OR: Odds ratio
